# Bronchiectasis morphology and hemoptysis recurrence after bronchial artery embolization: a cohort study using inverse probability of treatment weighting

**DOI:** 10.3389/fmed.2025.1645331

**Published:** 2025-09-12

**Authors:** Yuxin Duan, Weifan Sui, Zefeng Cai, Yimao Xia, Jianyun Li, Jianhua Fu

**Affiliations:** Department of Interventional Radiology, The Affiliated People's Hospital of Jiangsu University, Zhenjiang, Jiangsu, China

**Keywords:** bronchiectasis, hemoptysis, bronchial artery embolization, prognosis, morphological subtype, inverse probability of treatment weighting, recurrence

## Abstract

**Background:**

Hemoptysis is a serious and often life-threatening symptom associated with various pulmonary conditions, with bronchiectasis being a common cause. Bronchial artery embolization (BAE) is an effective intervention for managing hemoptysis, yet recurrence rates remain high. Several studies have identified general risk factors for recurrence. However, the role of bronchiectasis subtypes in predicting long-term outcomes after BAE has not been thoroughly explored.

**Objective:**

To assess the association between cystic bronchiectasis and the risk of hemoptysis recurrence following BAE, compared to non-cystic bronchiectasis subtypes.

**Methods:**

This retrospective cohort study included 168 adults treated with BAE for hemoptysis due to bronchiectasis from January 2017 to April 2022. Bronchiectasis was classified as cystic or non-cystic according to the Reid morphological criteria. Baseline differences between groups were adjusted using inverse probability of treatment weighting (IPTW).

**Results:**

Patients with cystic bronchiectasis exhibited a significantly higher recurrence risk than those with non-cystic bronchiectasis before and after IPTW adjustment (HR = 2.62; 95% CI, 1.42–4.82; *p* < 0.001). This association remained consistent across subgroup analyses stratified by age, sex, and various comorbidities, with cystic bronchiectasis consistently showing a higher recurrence risk.

**Conclusions:**

Cystic bronchiectasis is a significant independent predictor of hemoptysis recurrence following BAE. These findings suggest bronchiectasis morphology, especially cystic changes, should be recognized as an important prognostic factor when selecting candidates for BAE and planning long-term management.

## Introduction

Hemoptysis is a potentially life-threatening symptom associated with various pulmonary diseases. It poses a significant clinical challenge due to risks of asphyxiation and acute hemorrhage. Among its underlying causes, bronchiectasis is highly prevalent. Recurrent airway infections associated with bronchiectasis lead to structural damage and bleeding episodes in approximately 23%−52% of affected individuals (1–6). In China, up to 70% of bronchiectasis patients experience hemoptysis, significantly burdening healthcare resources (7). Bronchial artery embolization (BAE) is widely accepted as an effective intervention for controlling hemoptysis (8–10). Nevertheless, recurrence after BAE remains common, with reported recurrence rates ranging from 10 to 57% (11).

Several studies have identified general risk factors for post-BAE recurrence in bronchiectasis (5), however, the impact of bronchiectasis subtypes classified according to the Reid system (12)—on long-term outcomes remains insufficiently explored. Patients with cystic bronchiectasis notably exhibit higher recurrence rates and poorer prognoses compared to those with cylindrical or varicose types (7, 13). To further investigate this issue, a retrospective cohort study was conducted to determine if cystic bronchiectasis is associated with an increased risk of hemoptysis recurrence after BAE compared with non-cystic bronchiectasis.

## Methods

This retrospective study was approved by the Institutional Ethics Committee of the Affiliated People's Hospital of Jiangsu University (Ethical review no. SQK-2025055-W). The committee waived the requirement for informed consent. All BAE procedures were performed according to the guidelines of the Society of Interventional Radiology (14). The study was conducted in compliance with the ethical standards of institutional and national research committees. It also adhered to the Declaration of Helsinki (1964) and its subsequent amendments or equivalent ethical standards.

### Study participants

**Figure 1:**
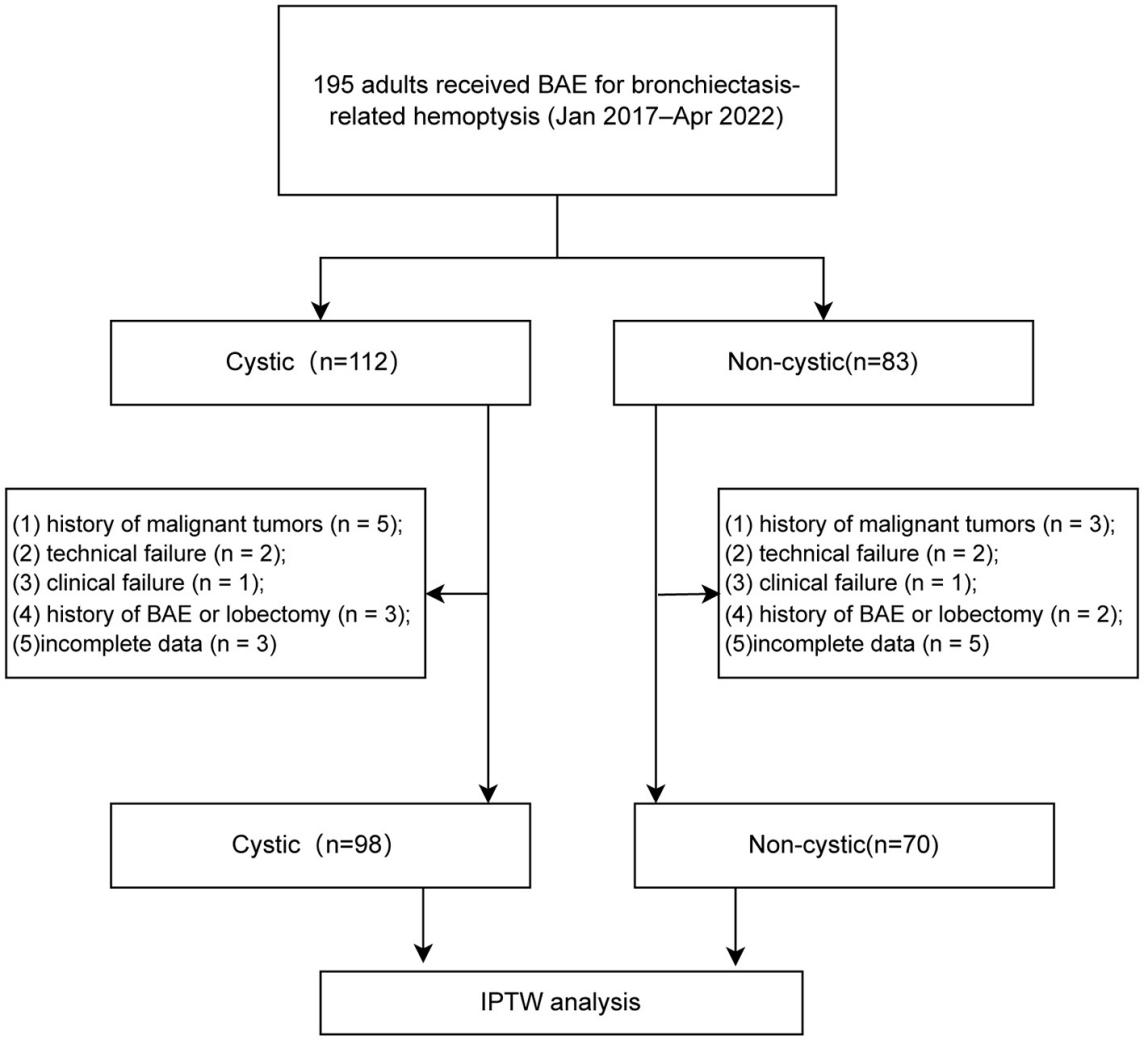
Flowchart for the selection procedure for bronchiectasis patients with hemoptysis treated by bronchial artery embolization. BAE, bronchial artery embolization; IPTW, inverse probability of treatment weighting.

Baseline information, preprocedural laboratory results, chest computed tomography (CT), bronchial artery CT angiography (CTA), and angiographic data of 195 adult patients were analyzed. These patients consecutively underwent arterial embolization for bronchiectasis-related hemoptysis at our institution from January 2017 to April 2022. The exclusion criteria included: (1) history of malignant tumors (*n* = 8), (2) technical failure (*n* = 4), (3) clinical failure (*n* = 2), (4) previous BAE or lobectomy (*n* = 5), and (5) incomplete data (*n* = 8). Ultimately, 168 patients were included in the study. The patient selection process is illustrated in Figure 1. Bronchiectasis was categorized into cystic or non-cystic (cylindrical or varicose) subtypes based on the Reid morphological classification and three experienced radiologists independently performed the classification.

### Data collection

The dataset included clinical profiles, preprocedural peripheral blood results, and imaging characteristics. Severity of hemoptysis was classified based on daily blood loss volume as mild (≤100 ml), moderate (100–300 ml), or severe (>300 ml), according to previously established criteria (5). Peripheral blood samples were obtained prior to embolization. A positive sputum culture was defined as detection of any pathogenic microorganism in sputum samples collected before BAE, following standard microbiological procedures (15). Pleural thickening >3 mm on CT was considered pathological (16). Hemoptysis history refers to hemoptysis occurring within 6 months before the current admission.

All patients underwent high-resolution chest CT and bronchial artery CTA before BAE to evaluate pulmonary infection and delineate potential culprit vessels for precise intraoperative targeting. The indications and procedural steps adhered to the guidelines of the Society of Interventional Radiology (17). Each procedure was performed under local anesthesia via right femoral arterial access. Initial visualization of the thoracic aortic branches was performed using a 5F pigtail catheter. Selective angiography of suspected culprit vessels, including bronchial and non-bronchial systemic arteries (NBSAs), was performed using either a 5F Cobra 3 catheter or a 5F left gastric catheter. Culprit vessels were identified based on angiographic features including vessel dilatation, tortuosity, hypervascularity, bronchial-pulmonary shunts, and contrast extravasation. Embolization was conducted using 2.7F or 2.4F microcatheters (Terumo Medical Corp., Japan; Boston Scientific, USA), and embolic agents were selected based on vessel caliber. These embolic agents included 350–560 μm polyvinyl alcohol (PVA) particles and gelatin sponge particles (GSP) (both from Hangzhou Alicon Pharmaceutical Co., Ltd., Zhejiang, China).

### Recurrence and follow-up

Clinical success was defined as complete cessation of hemoptysis or reduction to minimal levels (<10 ml/day) after BAE. Clinical failure referred to recurrent hemoptysis or death due to bleeding within 24 h after the procedure (18). Technical success was defined as successful embolization of all abnormal vessels, whereas technical failure indicated incomplete embolization or inability to identify culprit arteries (18). Recurrence was defined as hemoptysis ≥30 ml/day, the need for repeat BAE or surgical intervention (e.g., lobectomy), or death caused by recurrent bleeding after initial clinical success (16). Follow-up was performed via telephone interviews or outpatient visits and supplemented with radiological examinations as necessary. Follow-up was conducted from the date of the initial BAE procedure until the first recurrence of hemoptysis or until December 2024, whichever occurred first.

### Statistical analysis

Only patients with complete follow-up data were included in the final analysis. Continuous variables are presented as means ± standard deviations (SD), while categorical variables are presented as frequencies with corresponding percentages. Categorical variables were compared using the χ^2^ test or Fisher's exact test, and continuous variables were compared using the *t*-test or Wilcoxon rank-sum test, as appropriate. To minimize potential confounding, inverse probability of treatment weighting (IPTW) was applied to adjust for baseline differences between the treatment groups. Standardized mean differences (SMDs) were calculated to assess balance between the groups; an SMD < 0.2 was considered acceptable according to previously reported criteria (19).

Hemoptysis-free survival (HFS) was estimated using the Kaplan–Meier method, and differences between groups were assessed with the log-rank test. Cox proportional hazards regression models were used for both univariate and multivariate analyses of factors associated with HFS. Variables with *p*-values < 0.05 in the univariate analysis were included in the multivariate model. Hazard ratios (HRs) and corresponding 95% confidence intervals (CIs) were reported. Subgroup analyses were performed using stratified Cox models, with forest plots generated to illustrate the relationship between bronchiectasis morphology and hemoptysis recurrence across clinical subgroups. Statistical analyses were performed using R software (version 4.3.2) for Windows. A two-tailed *p*-value < 0.05 was considered statistically significant.

## Results

### Patient characteristics

Baseline characteristics of the cohort (*N* = 168) are summarized in Table 1. Overall, 98 patients (58.3%) had cystic bronchiectasis and 70 (41.7%) had non-cystic disease; 105 (62.5%) were male. A total of 114 patients (67.9%) were ≥60 years of age, including 76 (77.6%) in the cystic group and 38 (54.3%) in the non-cystic group. The median HFS was 783 days in the cystic group vs. 1,349 days in the non-cystic group. A history of hemoptysis was documented in 77 patients (45.8%; cystic 51 [52.0%] vs. non-cystic 26 [37.1%]). Pleural thickening was observed in 147 patients (87.5%; cystic 93 [94.9%] vs. non-cystic 54 [77.1%]). Fifty-two patients (31.0%) reported prior smoking. NBSAs were identified in 35 patients (20.8%) and SPSs in 67 (39.9%). The prevalence of major comorbidities—including diabetes, hypertension, and coronary heart disease—did not differ materially between groups. Among the 52 patients (31.0%) with positive sputum cultures, *Pseudomonas aeruginosa* was the most frequent pathogen (*n* = 35, 67.3%), followed by *Klebsiella pneumoniae* (*n* = 14, 26.9%) and *Candida albicans* (*n* = 3, 5.8%).

**Table 1:** Baseline characteristics of patients with cystic and non-cystic.

**Variable**	**Levels**	**Overall**	**Cystic**	**Non-cystic**	***p*-Value**
Gender, *n* (p%)	Male	105.00 (62.50%)	54.00 (55.10%)	51.00 (72.86%)	0.019
	Female	63.00 (37.50%)	44.00 (44.90%)	19.00 (27.14%)	
Age, years, *n* (p%)	<60	54.00 (32.14%)	22.00 (22.45%)	32.00 (45.71%)	0.001
	>=60	114.00 (67.86%)	76.00 (77.55%)	38.00 (54.29%)	
Volume, *n* (p%)	Massive	4.00 (2.38%)	4.00 (4.08%)	0.00 (0.00%)	0.192
	Mild	125.00 (74.40%)	70.00 (71.43%)	55.00 (78.57%)	
	Moderate	39.00 (23.21%)	24.00 (24.49%)	15.00 (21.43%)	
Hemoptysis history, *n* (p%)	No	91.00 (54.17%)	47.00 (47.96%)	44.00 (62.86%)	0.056
	Yes	77.00 (45.83%)	51.00 (52.04%)	26.00 (37.14%)	
Smoking, *n* (p%)	No	116.00 (69.05%)	77.00 (78.57%)	39.00 (55.71%)	0.002
	Yes	52.00 (30.95%)	21.00 (21.43%)	31.00 (44.29%)	
HPN, *n* (p%)	No	121.00 (72.02%)	72.00 (73.47%)	49.00 (70.00%)	0.621
	Yes	47.00 (27.98%)	26.00 (26.53%)	21.00 (30.00%)	
CHD, *n* (p%)	No	155.00 (92.26%)	92.00 (93.88%)	63.00 (90.00%)	0.354
	Yes	13.00 (7.74%)	6.00 (6.12%)	7.00 (10.00%)	
Diabetes, *n* (p%)	Absent	150.00 (89.29%)	85.00 (86.73%)	65.00 (92.86%)	0.206
	Present	18.00 (10.71%)	13.00 (13.27%)	5.00 (7.14%)	
NBSAs, *n* (p%)	Absent	133.00 (79.17%)	68.00 (69.39%)	65.00 (92.86%)	<0.001
	Present	35.00 (20.83%)	30.00 (30.61%)	5.00 (7.14%)	
SPSs, *n* (p%)	Absent	101.00 (60.12%)	49.00 (50.00%)	52.00 (74.29%)	0.002
	Present	67.00 (39.88%)	49.00 (50.00%)	18.00 (25.71%)	
Pleural thickening, *n* (p%)	Absent	21.00 (12.50%)	5.00 (5.10%)	16.00 (22.86%)	<0.001
	Present	147.00 (87.50%)	93.00 (94.90%)	54.00 (77.14%)	
Embolic materials, *n* (p%)	PVA	47.00 (27.98%)	12.00 (12.24%)	35.00 (50.00%)	<0.001
	PVA + GSP	94.00 (55.95%)	69.00 (70.41%)	25.00 (35.71%)	
	GSP	27.00 (16.07%)	17.00 (17.35%)	10.00 (14.29%)	
Sputum culture, *n* (p%)	Negative	116.00 (69.05%)	61.00 (62.24%)	55.00 (78.57%)	0.024
	Positive	52.00 (30.95%)	37.00 (37.76%)	15.00 (21.43%)	
LOS, median (IQR)		9.00 (5.00)	9.00 (6.00)	8.50 (5.00)	0.575
Albumin, g/L median (IQR)		37.70 (4.80)	37.00 (5.70)	39.15 (3.80)	<0.001
WBC, 10^9^, median (IQR)		6.90 (4.00)	7.20 (4.00)	6.60 (3.90)	0.149
HFS, days, median (IQR)		1,174.5 (1,313.5)	783 (949)	1,349 (1,016)	<0.001

CHD, coronary heart disease; HPN, hypertension; SPSs, systemic-pulmonary artery shunts; NBSAs, non-bronchial systemic arteries; WBC, white blood cell count; PVA, polyvinyl alcohol; GSP, gelatin sponge particles; HFS, hemoptysis-free survival.

### IPTW Method

**Figure 2:**
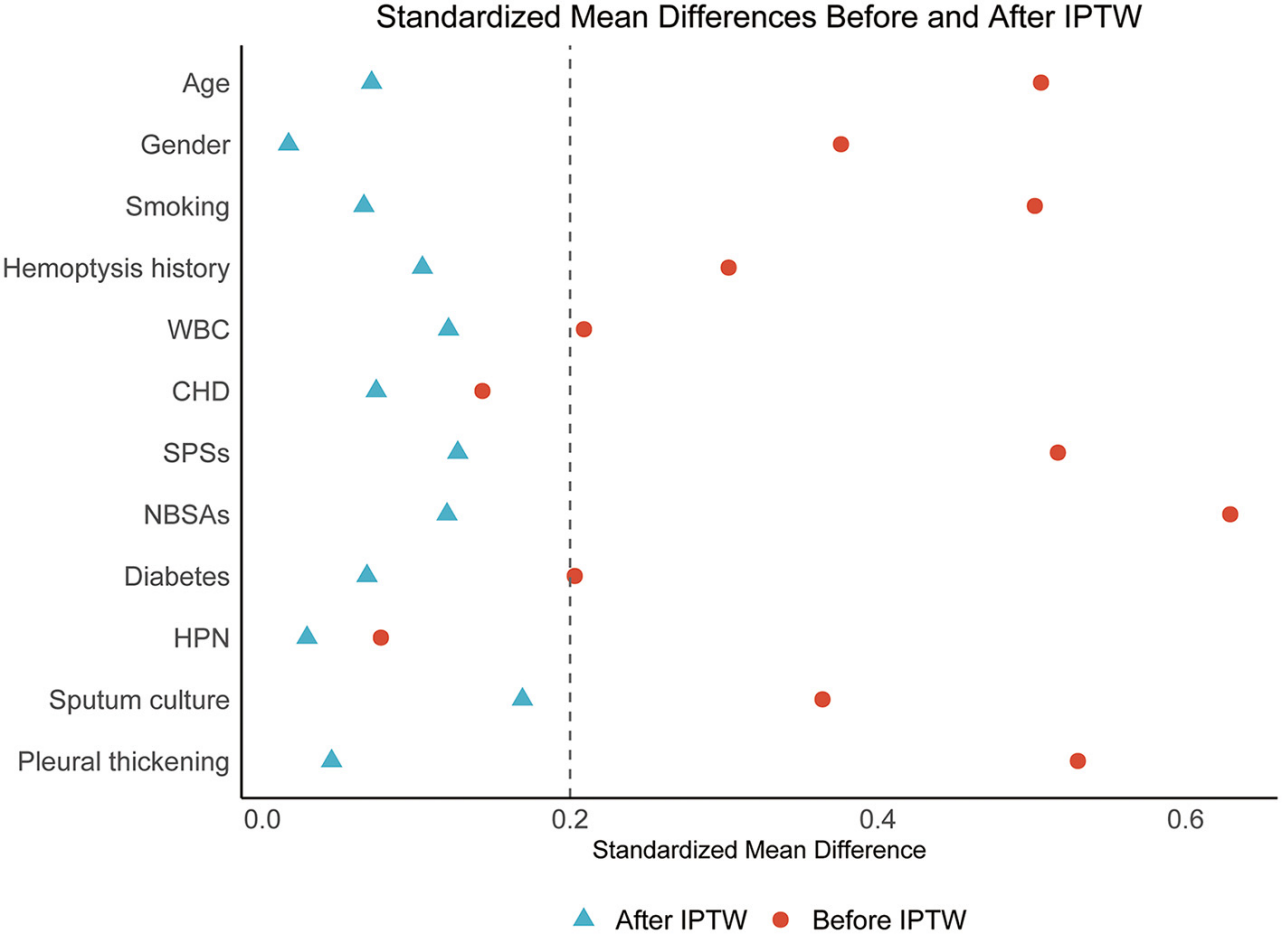
Love plot of standardized mean differences for selected baseline covariates before and after inverse probability of treatment weighting. IPTW, inverse probability of treatment weighting; CHD, coronary heart disease; HPN, hypertension; SPSs, systemic-pulmonary artery shunts; NBSAs, non-bronchial systemic arteries; WBC, white blood cell count.

IPTW was utilized to balance selected baseline covariates between cystic and non-cystic bronchiectasis groups, resulting in balanced and comparable cohorts (Table 2). The reduction in SMDs across confounders after IPTW adjustment is depicted in Figure 2.

**Table 2:** Baseline covariate distribution and standardized mean differences before and after IPTW.

**Variables**	**Before IPTW**				**After IPTW**			
	**Non-cystic group**	**Cystic group**	***p***	**SMD**	**Non-cystic group**	**Cystic group**	***p***	**SMD**
Age (≥60, %)	38 (54.3)	76 (77.6)	0.003	0.506	101.4 (66.5)	119.2 (69.8)	0.705	0.071
Gender (Female, %)	19 (27.1)	44 (44.9)	0.029	0.376	57.5 (37.7)	63.0 (36.9)	0.926	0.017
Smoking (Yes, %)	31 (44.3)	21 (21.4)	0.003	0.502	52.2 (34.2)	53.2 (31.1)	0.73	0.066
Hemoptysis history (Yes, %)	26 (37.1)	51 (52.0)	0.079	0.303	64.7 (42.4)	81.3 (47.6)	0.579	0.104
WBC (mean ± SD)	7.68 (2.91)	9.71 (13.38)	0.214	0.209	7.94 (3.24)	8.88 (10.43)	0.351	0.121
CHD (Yes, %)	7 (10.0)	6 (6.1)	0.526	0.143	11.4 (7.5)	9.6 (5.6)	0.635	0.074
SPSs (Present, %)	18 (25.7)	49 (50.0)	0.003	0.517	47.4 (31.1)	63.5 (37.1)	0.493	0.127
NBSAs (Present, %)	5 (7.1)	30 (30.6)	<0.001	0.629	24.1 (15.8)	34.9 (20.4)	0.572	0.12
Diabetes (Yes, %)	5 (7.1)	13 (13.3)	0.312	0.203	12.2 (8.0)	17.0 (10.0)	0.696	0.068
HPN (Yes, %)	21 (30.0)	26 (26.5)	0.749	0.077	37.5 (24.6)	39.8 (23.3)	0.86	0.029
Sputum culture (Positive, %)	15 (21.4)	37 (37.8)	0.037	0.364	39.7 (26.0)	57.6 (33.7)	0.38	0.169
Pleural thickening (Present, %)	54 (77.1)	93 (94.9)	0.001	0.53	130.5 (85.6)	143.4 (84.0)	0.828	0.045

IPTW, inverse probability of treatment weighting; SMD, standardized mean difference; CHD, coronary heart disease; HPN, hypertension; SPSs, systemic-pulmonary artery shunts; NBSAs, non-bronchial systemic arteries; WBC, white blood cell count.

### Kaplan–Meier analysis of HFS before and after IPTW adjustment

**Figure 3:**
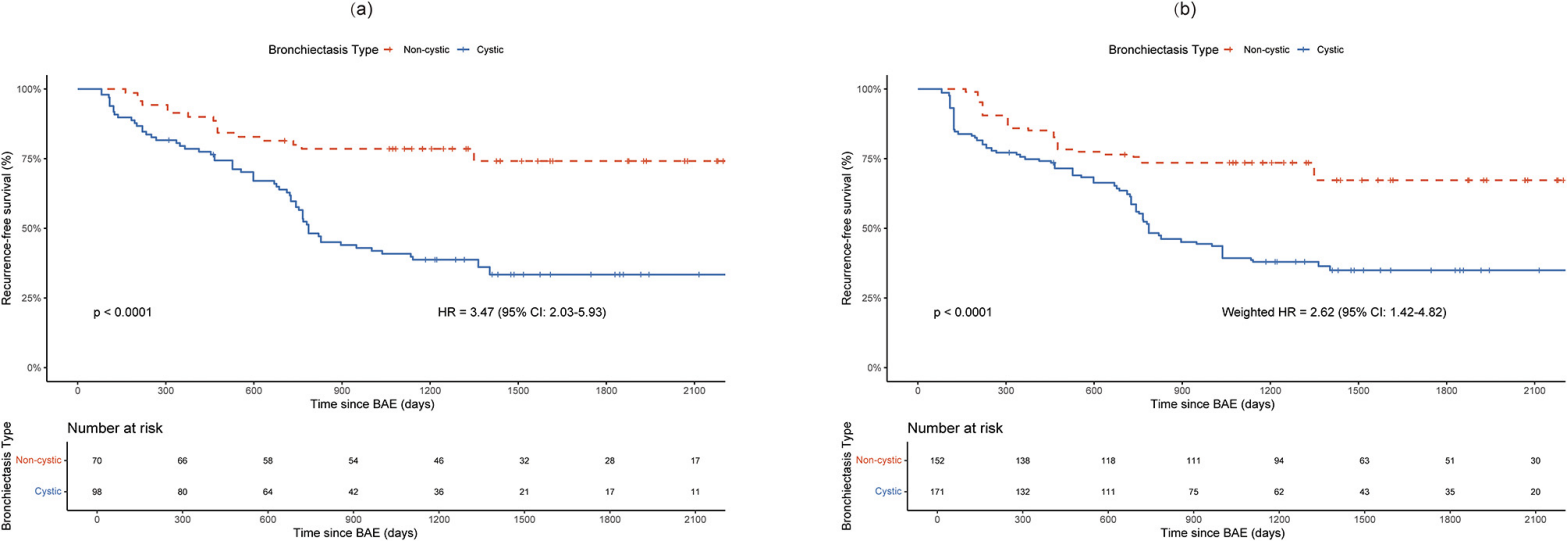
Kaplan–Meier curves for hemoptysis-free survival before **(a)** and after **(b)** inverse probability of treatment weighting. BAE, bronchial artery embolization; HR, hazard ratio; CI, confidence interval.

Kaplan–Meier analyses were performed to compare hemoptysis-free survival (HFS) between patients with cystic and non-cystic bronchiectasis before and after IPTW adjustment (Figure 3). In both unadjusted and IPTW-adjusted cohorts, patients with cystic bronchiectasis experienced significantly shorter HFS than those with non-cystic bronchiectasis (both *p* < 0.001). The unadjusted HR for recurrence in the cystic group was 3.47 (95% CI, 2.03–5.93), as shown in Figure 3a, while the IPTW-adjusted HR was 2.62 (95% CI, 1.42–4.82), as shown in Figure 3b. These results highlight a persistent and statistically significant association between cystic morphology and an increased risk of hemoptysis recurrence in patients with bronchiectasis.

### Association between bronchiectasis morphology and hemoptysis recurrence: overall and subgroup analyses

**Figure 4:**
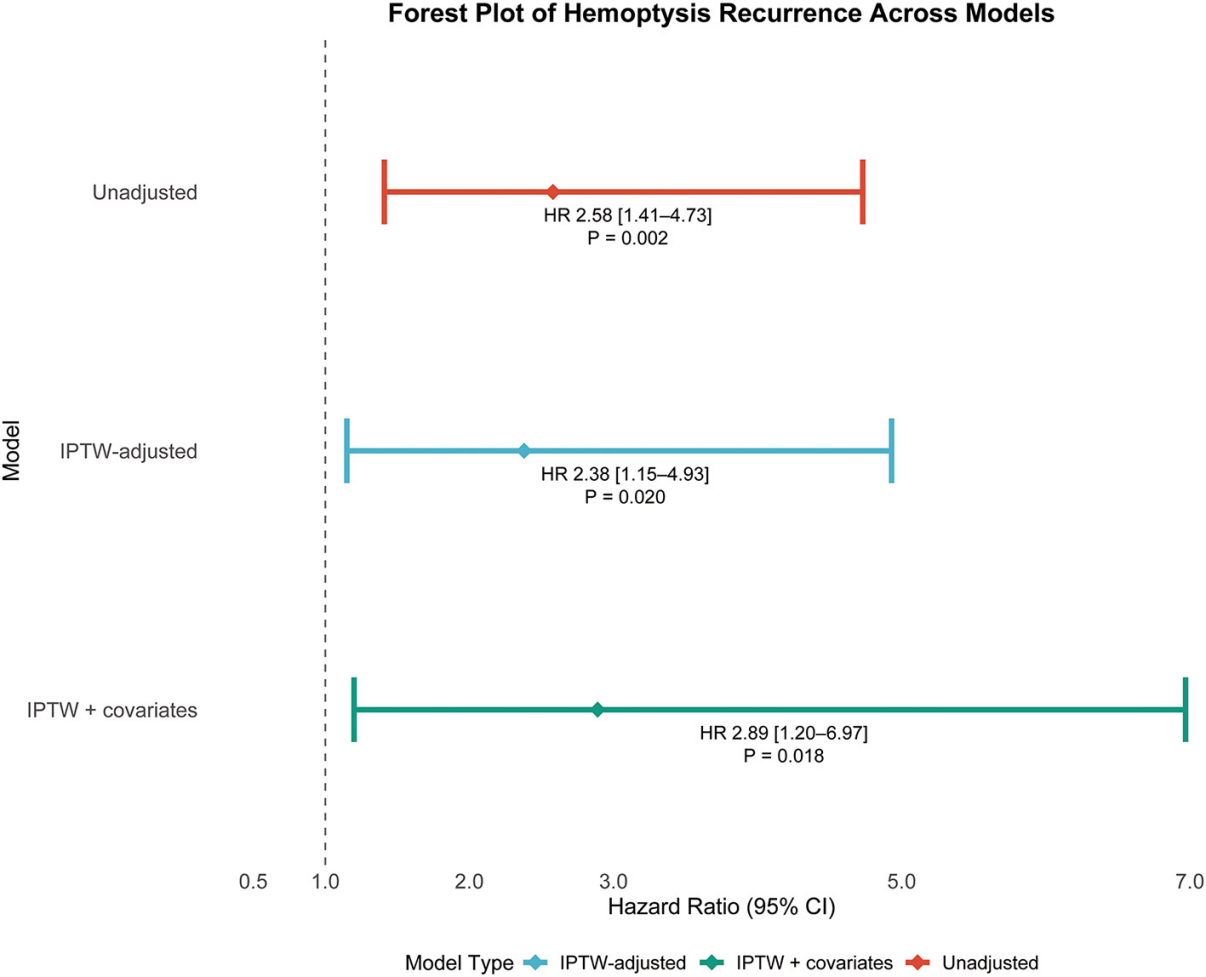
Forest plot depicting hazard ratios with 95% confidence intervals and associated *p*-values for recurrent hemoptysis in patients with cystic bronchiectasis across multiple statistical models. HR, hazard ratio; CI, confidence interval; IPTW, inverse probability of treatment weighting.

**Figure 5:**
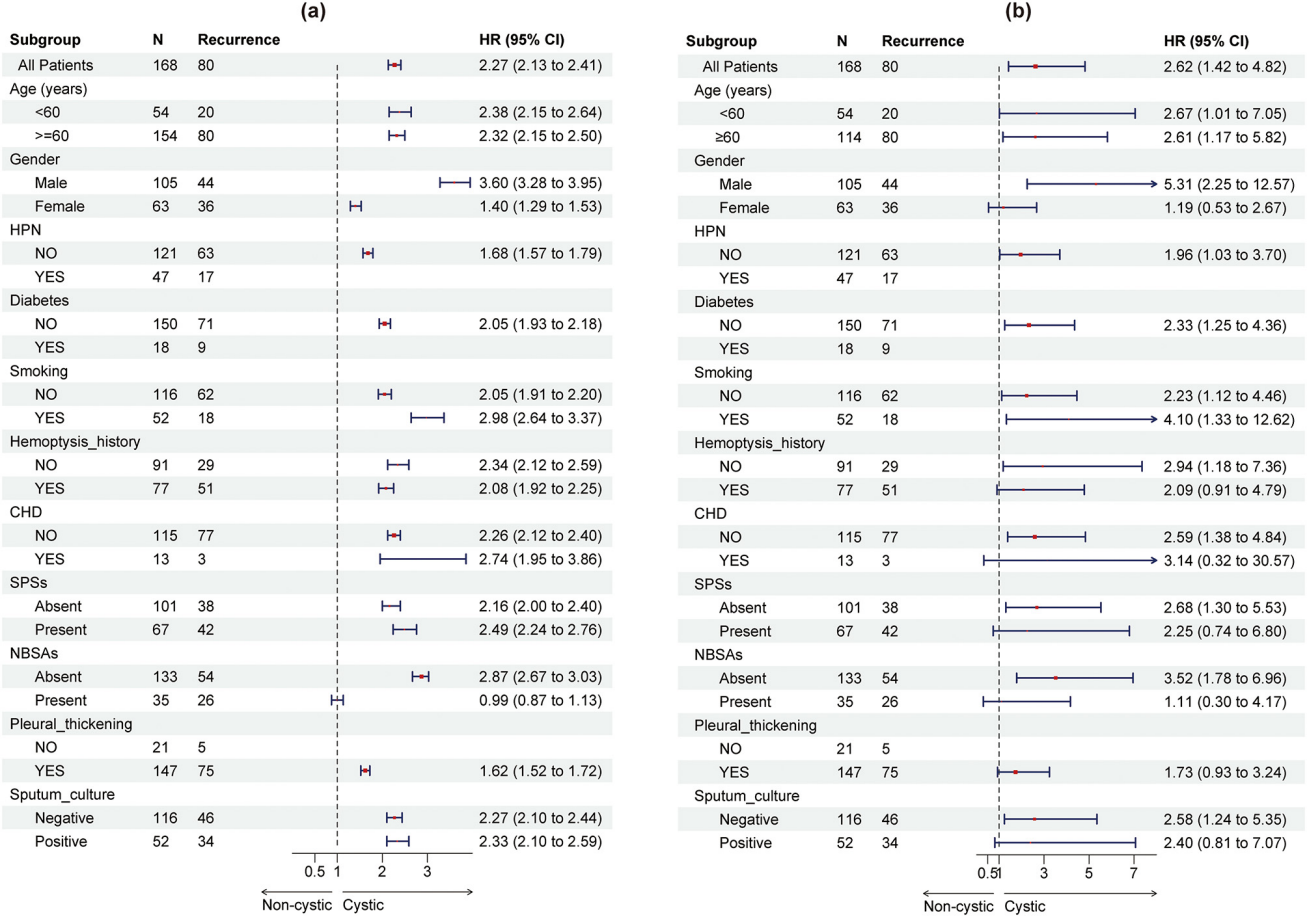
Subgroup-forest plots comparing hemoptysis recurrence risk between cystic and non-cystic bronchiectasis, before **(a)** and after **(b)** inverse probability of treatment weighting adjustment. CHD, coronary heart disease; HPN, hypertension; SPSs, systemic-pulmonary artery shunts; NBSAs, non-bronchial systemic arteries.

Multivariable Cox proportional hazards models were constructed to evaluate the association between bronchiectasis morphology (cystic vs. non-cystic) and the risk of hemoptysis recurrence under three analytical scenarios: (1) before weighting (HR, 2.58; 95% CI, 1.41–4.73; *p* = 0.002), (2) after IPTW adjustment with selected baseline covariates (HR, 2.38; 95% CI, 1.15–4.93; *p* = 0.020), and (3) after IPTW adjustment further including residual baseline variables (HR, 2.89; 95% CI, 1.20–6.97; *p* = 0.018), as detailed in Table 3. In all three models, cystic bronchiectasis consistently remained a statistically significant predictor of hemoptysis recurrence. The corresponding hazard ratios and confidence intervals are visually summarized in a forest plot (Figure 4). Patients with cystic bronchiectasis consistently demonstrated an elevated risk of hemoptysis recurrence across most analyzed subgroups, both before and after IPTW adjustment, as illustrated in Figures 5a, b.

**Table 3:** Univariate and multivariate analyses for predictive factors of HFS.

**Characteristic**	**Univariate**			**Multivariate**		
	**HR**	**95% CI**	***p*** **-Value**	**HR**	**95% CI**	***p*** **-Value**
TOB (Cystic)	2.62	1.42, 4.82	**0.002**	2.89	1.20, 6.97	**0.018**
Age (≥60 years)	1.04	0.57, 1.89	0.904	1.08	0.55, 2.14	0.815
Gender (Male)	1.53	0.90, 2.59	0.115	0.96	0.49, 1.90	0.918
Smoking (Yes)	0.64	0.35, 1.16	0.143	0.54	0.25, 1.17	0.117
Hemoptysis history (Yes)	2.81	1.59, 5.00	**< 0.001**	2.54	1.34, 4.79	**0.004**
WBC (10^9^)	1.00	1.00, 1.01	0.249	0.99	0.98, 1.01	0.323
CHD (Yes)	0.34	0.09, 1.19	0.091	0.36	0.07, 1.84	0.218
SPSs (Present)	0.34	0.09, 1.19	0.091	0.36	0.07, 1.84	0.218
NBSAs (Present)	1.80	1.01, 3.20	**0.047**	1.21	0.61, 2.42	0.580
Diabetes (Yes)	Yes	0.72	0.29, 1.77	0.473	1.54	0.52, 4.61
HPN (Yes)	0.43	0.24, 0.80	**0.007**	0.52	0.26, 1.05	0.066
Sputum culture (Positive)	1.84	1.08, 3.13	**0.024**	1.77	0.96, 3.28	0.069
Pleural thickening (Present)	0.68	0.30, 1.57	0.368	0.36	0.12, 1.05	0.062
Albumin (g/L)	1.00	0.94, 1.06	0.965	1.07	0.96, 1.19	0.197
**Embolic materials**						
PVA+GSP	2.13	1.05, 4.32	**0.036**	1.80	0.63, 5.11	0.269
GSP	2.04	0.71, 5.83	0.183	2.92	1.03, 8.28	**0.044**
LOS	1.01	0.96, 1.05	0.803	1.06	1.01, 1.12	**0.023**
**Volume**						
Moderate	1.14	0.61, 2.11	0.684	0.93	0.42, 2.08	0.862
Massive	2.54	0.89, 7.23	0.080	0.83	0.27, 2.51	0.736

TOB, type of bronchiectasis; CHD, coronary heart disease; HPN, hypertension; SPSs, systemic-pulmonary artery shunts; NBSAs, non-bronchial systemic arteries; WBC, white blood cell count; PVA, polyvinyl alcohol; GSP, gelatin sponge particles.

Bold values indicate statistical significance at *p* < 0.05.

## Discussion

Although BAE achieves high initial success rates (70%−99%) in managing acute or recurrent hemoptysis, the long-term recurrence remains substantial and has improved little over time. Previous research suggests that cystic bronchiectasis may be associated with poorer outcomes compared with non-cystic bronchiectasis. However, few studies have systematically evaluated the prognostic significance of bronchiectasis morphology, as classified by Reid, in predicting recurrence of hemoptysis following BAE. This study, leveraging a relatively large sample size and utilizing IPTW to minimize confounding bias, systematically demonstrated that cystic bronchiectasis significantly increases the risk of hemoptysis recurrence after BAE.

A primary limitation of this retrospective study is the imbalance in baseline characteristics between patient groups. To reduce confounding bias, we excluded patients with contraindications to BAE, malignant tumors, and impaired cardiovascular function. Nonetheless, initial analysis still revealed imbalanced baseline characteristics between the two groups. To address this issue, IPTW-based analysis was performed. The SMDs obtained from IPTW analysis confirmed effective balancing of the selected confounders between the two groups (20).

Unlike previous studies, which mainly relied on small-scale subgroup analyses to compare cystic vs. non-cystic bronchiectasis (7, 13), our study—featuring a larger cohort and a more rigorous methodology—provides a more robust understanding of the association between bronchiectasis morphology and hemoptysis recurrence. We found that patients with cystic bronchiectasis had a significantly higher risk of hemoptysis recurrence compared with those with non-cystic bronchiectasis (HR, 2.38; 95% CI, 1.15–4.93), consistent with previously reported hazard ratios of 2.79 (95% CI, 1.12–6.96) and 1.52 (95% CI, 1.10–6.72). This finding may partly be explained by the distinct pathomorphological characteristics of cystic bronchiectasis. As described by King (21), cystic bronchiectasis is characterized by severe bronchial wall destruction and impaired mucociliary clearance, facilitating mucus retention and persistent bacterial colonization. These alterations predispose patients to recurrent infections and chronic airway inflammation. The resulting persistent inflammatory state compromises the structural integrity of the airways and promotes neovascularization in bronchial and peribronchial tissues. According to Lu et al. (13), these newly formed vessels are typically fragile and prone to rupture, especially during episodes of acute inflammation or elevated airway pressures. These mechanisms likely contribute to both the initial occurrence and subsequent recurrence of hemoptysis following BAE.

The robustness of the association between cystic bronchiectasis and recurrent hemoptysis was consistently demonstrated across multiple analytic approaches, including unweighted analysis, IPTW, and IPTW combined with covariate-adjusted Cox regression models. Hazard ratios remained statistically significant across all analytic models (HRs, 2.58, 2.38, and 2.89; all *p* < 0.05) as shown in Figure 4, reinforcing the conclusion that cystic bronchiectasis morphology is a robust and independent predictor of hemoptysis recurrence.

Furthermore, we examined this association in clinically relevant subgroups. Subgroup analyses, both before and after IPTW adjustment, demonstrated that patients with cystic bronchiectasis consistently exhibited a higher risk of hemoptysis recurrence compared with those having non-cystic bronchiectasis across most subpopulations, including those stratified by age, sex, comorbidities, and prior hemoptysis history. These findings emphasize the generalizability of this association, suggesting that cystic bronchiectasis morphology confers an elevated risk across diverse clinical populations.

In clinical practice, prognostic factors—including the presence of NBSAs and SPSs—significantly affect patient outcomes (22–24). Multivariate analysis identified three independent predictors of shorter HFS: a history of hemoptysis within 6 months before admission, exclusive use of GSP during BAE, and prolonged hospitalization. A history of hemoptysis typically indicates chronic, severe bronchiectasis accompanied by complex vascular remodeling and collateral formation (25). Exclusive use of GSP during embolization was also linked to a higher risk of recurrent hemoptysis in our study. As a temporary embolic agent, GSP is prone to early recanalization, potentially resulting in incomplete and transient vessel occlusion. However, when bronchial arteries are highly tortuous or difficult to superselect distally, GSP is preferred to minimize non-target embolization risks associated with permanent agents like PVA. This technical limitation partly explains the selection of GSP in certain patients, but may contribute to the increased recurrence rates observed in previous studies (5). Additionally, prolonged hospitalization increases the risk of nosocomial infections, potentially exacerbating underlying pulmonary conditions and thus raising the likelihood of recurrent hemoptysis, consistent with previous reports (26).

Several limitations should be acknowledged in this study. First, this study was conducted at a single center using a retrospective cohort design; despite applying IPTW and multivariable adjustments, residual bias may remain. Second, limited sample size precluded subclassification of non-cystic bronchiectasis into cylindrical and varicose types, potentially obscuring heterogeneity in recurrence risk within this group. Third, procedural factors—including variations in embolization technique, operator expertise, and embolic material type—were not controlled, potentially affecting recurrence outcomes. Lastly, although subgroup analyses were conducted, small sample sizes in some strata may have limited statistical power to detect interaction effects.

In conclusion, this retrospective cohort study demonstrates a significant association between cystic bronchiectasis and increased recurrence risk of hemoptysis following bronchial artery embolization. This association remained robust in various statistical models and was consistently observed across diverse clinical subgroups. These findings indicate bronchiectasis morphology, particularly cystic changes, as a critical prognostic factor in patient evaluation and long-term management planning following BAE. Further prospective, multicenter studies with larger sample sizes and standardized morphological classifications are warranted to validate these findings and refine individualized management strategies for patients experiencing bronchiectasis-related hemoptysis.

## Data Availability

The raw data supporting the conclusions of this article will be made available by the authors, without undue reservation.
